# Supplementary material for: The causal role of circulating inflammatory markers in osteoporosis: a bidirectional Mendelian randomized study
Source: Front Immunol. 2024 Jul 18;15:1412298. doi: 10.3389/fimmu.2024.1412298 (PMC11291241; doi:10.3389/fimmu.2024.1412298)
Supplement: Supplementary file 1 [file DataSheet_1.zip › supplementary material/OSTEOPOROSIS-CIM/GCST90274773.tsv.gz_forest.pdf]

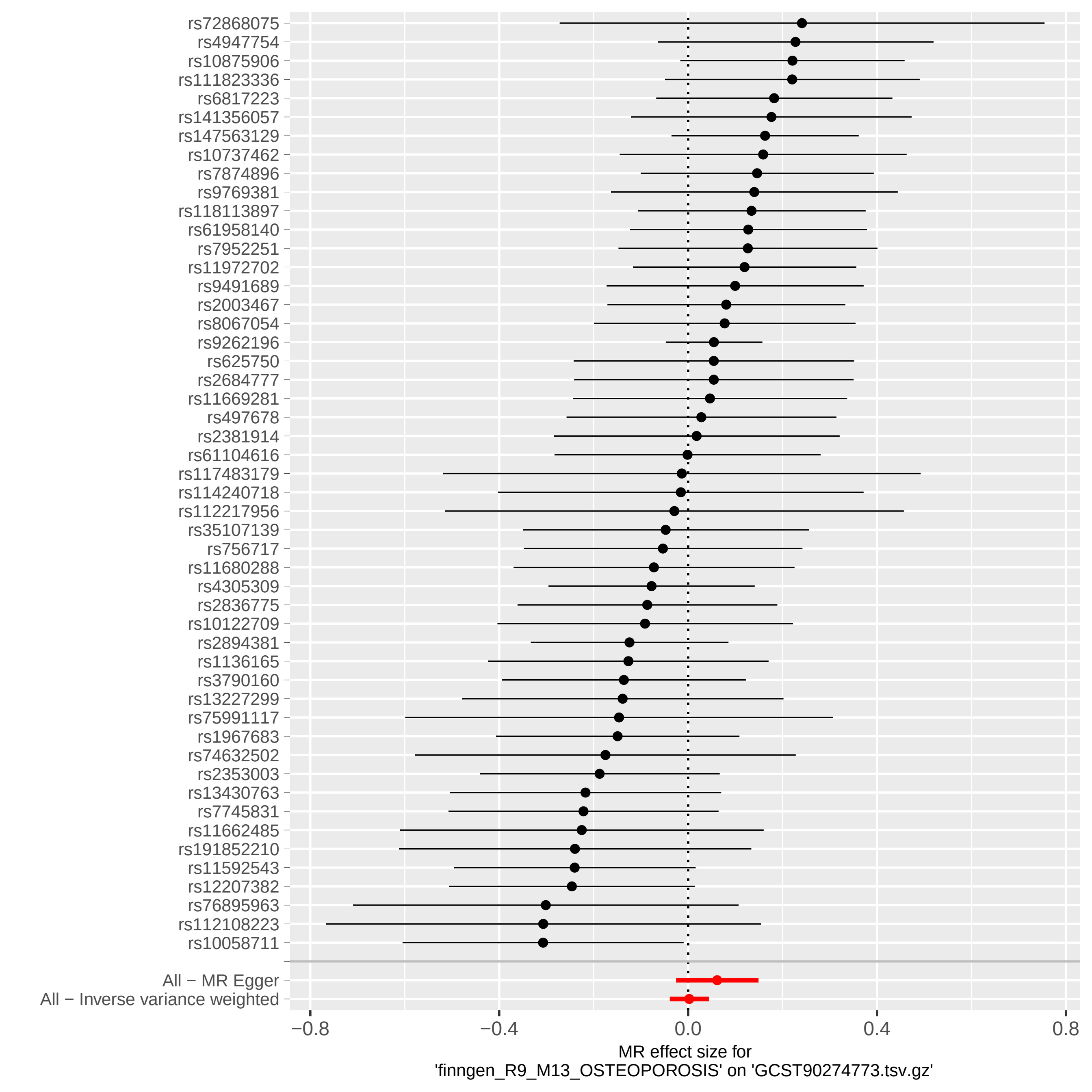
rs72868075
rs4947754
rs10875906
rs111823336
rs6817223
rs141356057
rs147563129
rs10737462
rs7874896
rs9769381
rs118113897
rs61958140
rs7952251
rs11972702
rs9491689
rs2003467
rs8067054
rs9262196
rs625750
rs2684777
rs11669281
rs497678
rs2381914
rs61104616
rs117483179
rs114240718
rs112217956
rs35107139
rs756717
rs11680288
rs4305309
rs2836775
rs10122709
rs2894381
rs1136165
rs3790160
rs13227299
rs75991117
rs1967683
rs74632502
rs2353003
rs13430763
rs7745831
rs11662485
rs191852210
rs11592543
rs12207382
rs76895963
rs112108223
rs10058711
All − MR Egger
All − Inverse variance weighted
−0.8
−0.4
0.0
0.4
0.8
MR effect size for
'finngen_R9_M13_OSTEOPOROSIS' on 'GCST90274773.tsv.gz'
